# Opioid prescribing patterns among medical practitioners in New South Wales, Australia

**DOI:** 10.1111/dar.13675

**Published:** 2023-05-09

**Authors:** Andrea L. Schaffer, Natasa Gisev, Fiona M. Blyth, Nicholas A. Buckley, David Currow, Timothy A. Dobbins, Andrew Wilson, Louisa Degenhardt, Sallie‐Anne Pearson

**Affiliations:** ^1^ School of Population Health Faculty of Medicine and Health, UNSW Sydney Sydney Australia; ^2^ National Drug and Alcohol Research Centre, UNSW Sydney Sydney Australia; ^3^ School of Public Health, Faculty of Medicine and Health The University of Sydney Sydney Australia; ^4^ Biomedical Informatics and Digital Health, Faculty of Medicine and Health The University of Sydney Sydney Australia; ^5^ Faculty of Science, Medicine and Health University of Wollongong Wollongong Australia; ^6^ Menzies Centre for Health Policy and Economics, Faculty of Medicine and Health The University of Sydney Sydney Australia

**Keywords:** opioids, pain, Australia, pharmacoepidemiology, prescribing

## Abstract

**Introduction:**

Prescriber behaviour is important for understanding opioid use patterns. We described variations in practitioner‐level opioid prescribing in New South Wales, Australia (2013–2018).

**Methods:**

We quantified opioid prescribing patterns among medical practitioners using population‐level dispensing claims data, and used partitioning around medoids to identify clusters of practitioners who prescribe opioids based on prescribing patterns and patient characteristics identified from linked dispensing claims, hospitalisations and mortality data.

**Results:**

The number of opioid prescribers ranged from 20,179 in 2013 to 23,408 in 2018. The top 1% of practitioners prescribed 15% of all oral morphine equivalent (OME) milligrams dispensed annually, with a median of 1382 OME grams (interquartile range [IQR], 1234–1654) per practitioner; the bottom 50% prescribed 1% of OMEs dispensed, with a median of 0.9 OME grams (IQR 0.2–2.6). Based on 63.6% of practitioners with ≥10 patients filling opioid prescriptions in 2018, we identified four distinct practitioner clusters. The largest cluster prescribed multiple analgesic medicines for older patients (23.7% of practitioners) accounted for 76.7% of all OMEs dispensed and comprised 93.0% of the top 1% of practitioners by opioid volume dispensed. The cluster prescribing analgesics for younger patients with high rates of surgery (18.7% of practitioners) prescribed only 1.6% of OMEs. The remaining two clusters comprised 21.2% of prescribers and 20.9% of OMEs dispensed.

**Discussion and Conclusion:**

We observed substantial variation in opioid prescribing among practitioners, clustered around four general patterns. We did not assess appropriateness but some prescribing patterns are concerning. Our findings provide insights for targeted interventions to curb potentially harmful practices.

**Key Points**

- We quantified for the first‐time variation in opioid prescribing among medical practitioners in a whole‐of‐population Australian cohort.
- There was substantial variation in opioid prescribing with the top centile prescribing many orders of magnitude more opioids than those in the bottom centiles.
- Practitioners were clustered around four general patterns of opioid prescribing behaviour and patient characteristics.

## 1 INTRODUCTION

Opioids are prescribed for a wide range of pain indications, including cancer, chronic non‐cancer pain, palliation and acute pain following surgery. In developed countries, there are concerns about opioid over‐prescribing, the drivers of which are multifactorial, including system, patient and practitioner‐level factors [1]. While there are many Australian studies of overall population‐level opioid prescribing and dispensing [2, 3], which have identified increases in opioid use over time, no large studies have quantified practitioner‐level prescribing behaviour. International data have found that individually, while specialists in fields such as anaesthetics and pain medicine prescribe a large volume of opioids, overall the greatest opioid volumes are prescribed in primary care [4, 5, 6]. In the United States, 20%–25% of all opioid prescriptions were written by family medicine providers, compared with 9%–10% by specialists in pain medicine [5, 7]. Opioid prescribing patterns are also highly skewed. A 2020 US study found that the top 1% of opioid prescribers accounted for 49% of all oral morphine equivalent (OME) milligrams prescribed and 27% of all opioid prescriptions [4]. In the United Kingdom, the total OME mgs prescribed varied almost eight‐fold by general practice [8].

Opioid prescribing behaviour is likely to vary greatly between countries due to differences in health system structures, medicine regulation, subsidy restrictions and local policies. In Australia, there has been no population‐based examination of practitioner‐level opioid prescribing patterns. In this study, we used data on all adult New South Wales (NSW) residents initiating opioids to: (i) describe the variation in practitioner‐level opioid prescribing; (ii) identify and characterise distinct practitioner clusters based on opioid prescribing and patient characteristics; and (iii) quantify the variation in opioid prescribing by practitioner cluster.

## 2 METHODS

### 2.1 Data source and study population

We used data from the POPPY II study [9] which includes linked medicine claims, hospitalisation and mortality registry data for adult NSW residents who initiated prescribed opioids between 2003 and 2018. Australian citizens and eligible residents receive subsidised access to medicines through the pharmaceutical benefits scheme (PBS). The PBS data capture data on all PBS‐listed medicines dispensed anywhere in Australia, and include information on the medicine dispensed, quantity supplied, date of dispensing, unique prescriber ID, as well as patient, pharmacy and prescriber postcode. These data do not capture medicines dispensed privately (where the patient pays the full cost out‐of‐pocket) or in public hospitals. The vast majority of opioids are dispensed through the PBS [10]. We included all NSW‐based medical practitioners who prescribed opioids to people residing in NSW between 2013 and 2018; we focussed on this time period as prior to 2013 complete capture of dispensing for PBS‐listed opioids was not available [11].

We excluded dentists, nurse practitioners, midwives and ophthalmologists as they prescribe a very small proportion of opioids relative to medical practitioners [12]. We did not have any further information on practitioner specialty to differentiate between practitioner types (e.g., general practitioners and pain specialists). We excluded practitioners who were based outside of NSW for all or part of each year; this was determined using data on practitioner postcodes and the postcode of dispensing pharmacies. Only one postcode is recorded per practitioner; however, a small number of practitioners may practice in multiple states. Thus, to address potential under‐capture of prescribing for these individuals, we excluded practitioners where all patients were dispensed prescriptions from non‐NSW pharmacies (Figure S1, Supporting Information).

To characterise practitioners based on characteristics of their patients, we also used data from the National Death Index, which records all deaths in Australia, and hospitalisation data from the NSW Admitted Patient Data Collection. The Admitted Patient Data Collection captures all admitted patient services provided by public and private hospitals and includes diagnoses (International Statistical Classification of Diseases and Related Health Problems, Tenth Revision, Australian Modification) and procedures performed (Australian Classification of Health Interventions). Primary care data (including outpatient diagnoses) are not routinely collected in Australia, and thus we relied on inpatient diagnoses, medicine dispensings and mortality data to characterise patients' health characteristics.

### 2.2 Opioid prescribing

We used dispensing claims of PBS‐listed opioids for analgesia to measure practitioner‐level prescribing practices, these were: buprenorphine, codeine, codeine + paracetamol, codeine + aspirin, fentanyl, hydromorphone, methadone, morphine, oxycodone, oxycodone + naloxone, tapentadol and tramadol. Opioid agonist treatment for opioid dependence (e.g., methadone, buprenorphine) were identified by their PBS item code and excluded. We calculated the volume of opioids dispensed using OME milligrams and grams [13]. We did not have information on opioids prescribed but not dispensed, nor privately dispensed medicines. Further, our data do not record the specific indication for prescribing.

For each year of the study period (2013–2018), we summed the number of opioid dispensings, number of patients prescribed opioids and total OME mgs dispensed to patients per practitioner. We used the Lorenz curve to graphically assess the skewness of the extent of opioid prescribing by different practitioners [14]. Prescribers were ranked in descending order of opioid prescribing in OME mgs, and we calculated the total number of OME mgs for the top 1%, 10% and 50% of practitioners in each year. This is a common method for quantifying variation in prescribing behaviour [4, 14].

### 2.3 Cluster analysis

To better understand drivers of practitioner‐level opioid prescribing, we performed a cluster analysis on the subset of practitioners whose patients were dispensed opioids in 2018. We included practitioner‐level prescribing characteristics and also aimed to describe the types of patients treated by a given practitioner by including patient characteristics that may influence opioid prescribing. All practitioner‐ and patient‐level characteristics were measured cross‐sectionally using data from 2018. The list of variables in the cluster analysis were: mean OME per patient; mean OME per dispensing; opioid dispensings (%) that were codeine, buprenorphine, tramadol, oxycodone, morphine or fentanyl; patients that were newly prescribed opioids (%); patients with one opioid dispensing (%); percentage of all dispensings that were for opioids; mean patient age; patients who died (%); patients with cancer (%) based on inpatient diagnoses or dispensing of anticancer medicines; patients with back pain, join pain or chronic pain based on inpatient diagnoses (%); patients hospitalised (%); median patient length of stay in hospital; patients who underwent a surgical procedure (%); patients prescribed antidepressants, benzodiazepines, pregabalin or nonsteroidal anti‐inflammatory drugs (%); and total number of medicine classes prescribed. Inpatient diagnoses were obtained from hospitalisation data (Admitted Patient Data Collection), while prescribing data was obtained from the PBS. Table S1 details the full list of variables and their definitions, including the data source and relevant codes.

As we had a mix of categorial and continuous variables on different scales, we used partitioning around medoids, an application of the k‐medoids clustering algorithm, to identify groups of practitioners [15]. The k‐medoids algorithm places practitioners into clusters, where the centre is represented by an observation in the cluster (called the ‘medoid’). This algorithm minimises the dissimilarity between each medoid and all practitioners in that cluster, and is less influenced by outliers or noise than k‐means. As some variables were on different scales, they were normalised to the range 0 to 1 and log transformed as necessary prior to clustering. Statistical metrics (Silhouette method, Gap statistic) [15] and clinical judgement were used to select the final number of clusters of practitioners. In previous work, results from distance‐based algorithms and model‐based approaches tend to find similar profiles [16, 17].

### 2.4 Sensitivity analysis

As we may be missing dispensing data on practitioners who moved interstate or stopped practising partway through a year and thus under‐estimating the volume of OMEs dispensed, we recalculated the Lorenz curve and opioid dispensing distribution in practitioners with at least one NSW‐based patient who was dispensed any prescription in each quarter of the year. Secondly, we verified the consistency of the clusters derived over time by repeating the analysis in 2016 and 2017; we did not repeat the cluster analysis in earlier years due to changes in recording of comorbidities in hospital separation data.

### 2.5 Ethics approval

The study protocol has received full ethical approval from the Australian Institute of Health and Welfare Ethics Committee (EO2016/4/314), NSW Population and Health Services Research Committee (2017/HRE0208), the ACT Health Human Research Ethics Committee (ETHLR.18.094) and the ACT Calvary Public Hospital Bruce Ethics Committee (5‐2019).

## 3 RESULTS

We identified 32,876 NSW medical practitioners who prescribed opioids over the 6‐year study period, comprising 134,128 practitioner‐years. The number of practitioners ranged from 20,179 in 2013 to 23,408 in 2018. This compares with 31,269 to 36,194 medical practitioners registered with the Australian Health Practitioner Regulation Agency in NSW between 2013/2014 and 2017/2018 (Table S2) [18]. Nearly half of practitioners (*n* = 13,485; 41.0%) prescribed opioids in all 6 years, while 15% prescribed opioids in 1 year only; the median was 5 years of prescribing (interquartile range [IQR] 2–6).

Over the entire 6‐year period, practitioners prescribed opioids to a median of 21 patients per year (IQR 4–71). The median number of opioid dispensings per practitioner was 32 per year (IQR 5–175), and the median OME mgs per practitioner was 8125 per year (IQR 890–92,963). These estimates were observed consistently over all study years (Table S3).

### 3.1 Distribution of opioid prescribing

**FIGURE 1:**
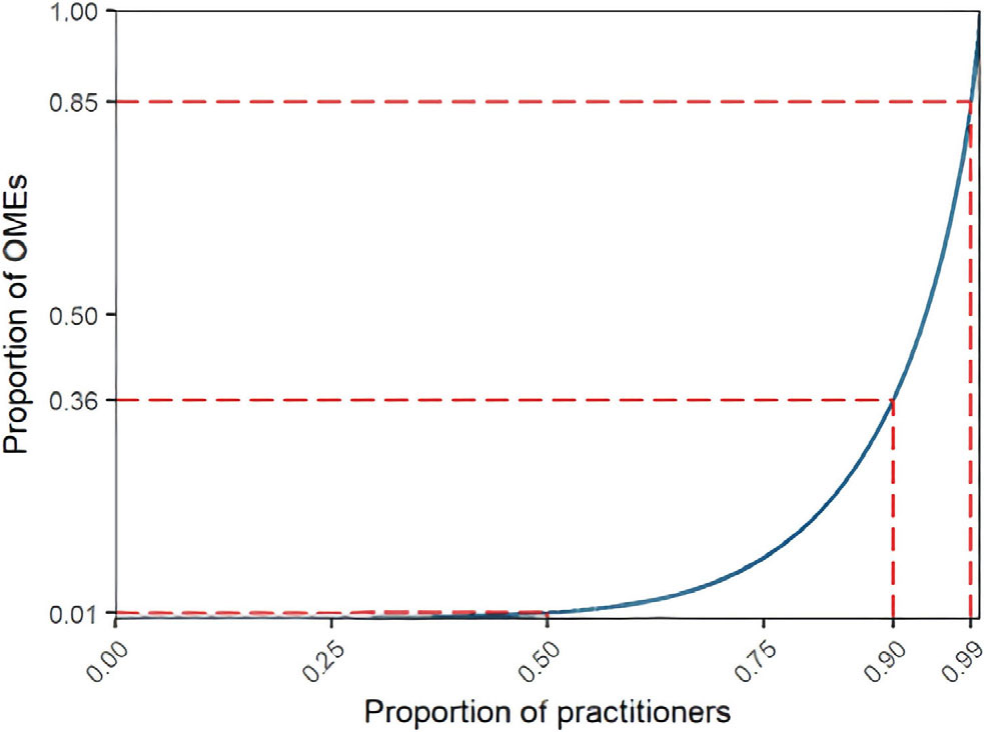
Lorenz curve showing the distribution of oral morphine equivalents (OME) mgs prescribed in 2018 represented by the blue line (*n* = 23,408). The dashed red lines show the proportion of OME mgs prescribed by the top 50%, 90%, and 99% of practitioners. If opioid prescribing were equally distributed among practitioners, the blue line would be straight with a slope of 1.

Figure 1 shows the Lorenz curve for total OME mgs prescribed in 2018. When ranked in descending order of opioid prescribing in OME mgs, the top 1% of practitioners (*n* = 329) prescribed 15.1% of all OME mgs dispensed annually, the top 10% (*n* = 3288) prescribed 63.9% and the bottom 50% (*n* = 16,438) prescribed 1%; again, these estimates were consistent across all study years (Figure S2 and Table S4).

The top 1% of practitioners prescribed a median of 1382 OME grams (IQR 1234–1654) per year, nearly triple the amount prescribed by the top 2%–10% of practitioners (505 OME grams, IQR 399–688). The bottom 50% prescribed only a median of 0.9 OME grams (IQR 0.2–2.6) (Table 1). The practitioners in the top 1% also prescribed opioids to a greater number of patients, with a median of 259 patients (IQR 199–328). While the median number of opioid dispensings per patient was similar across rankings, patients of practitioners in the top centile were prescribed more OMEs in total, and a greater number of OMEs per dispensing (Table 1).

**TABLE 1:** Patterns of opioid prescribing by opioid prescribing rank, 2013 to 2018 (*n* = 32,876). Practitioners who prescribed opioids in multiple years are counted more than once.

Prescribing patterns per year	Rank based on total OME (mg/g) prescribed per year			
	Top 1% of opioid prescribers (*n* = 1339)	Top 2%–10% of opioid prescribers (*n* = 12,072)	Top 11%–50% of opioid prescribers (*n* = 53,649)	Bottom 50% of opioid prescribers (*n* = 67,070)
	Median (IQR)	Median (IQR)	Median (IQR)	Median (IQR)
No. patients prescribed opioids per practitioner	259 (199–328)	151 (111–203)	55 (29–95)	4 (2–12)
No. opioid dispensings per practitioner	1491 (1121–1959)	668 (496–911)	127 (62–245)	6 (2–14)
OME grams per prescriber	1382 (1234–1654)	505 (399–688)	60 (23–140)	0.9 (0.2–2.6)
Opioid dispensings per patient, mean^a^	3 (2–3)	2 (2–3)	2 (1–2)	1 (1–1)
OME mgs per patient, mean^a^	902 (671–1217)	583 (418–811)	296 (190–461)	119 (76–182)
OME mgs per dispensing, mean^a^	451 (370–561)	360 (296–444)	242 (173–329)	109 (73–154)

Abbreviations: IQR, interquartile range; OME, oral morphine equivalent.

^a^ Mean Is calculated on log scale (geometric mean).

### 3.2 Clusters of distinct practitioner groups

We included two‐thirds of medical practitioners in 2018 who prescribed opioids to ≥10 patients (*n* = 14,874, 63.5%) in the cluster analysis. Table S5 details the four distinct clusters of practitioners based on their opioid prescribing patterns and patient characteristics. Table 2 includes the summary statistics for all variables in the cluster analysis by cluster.

**TABLE 2:** Distribution of clustering variables in 2018 in practitioners with 10+ patients (*n* = 14,874).

	Cluster 1 (*n* = 5536)	Cluster 2 (*n* = 4387)	Cluster 3 (*n* = 4013)	Cluster 4 (*n* = 938)
	Median, IQR	Median, IQR	Median, IQR	Median, IQR
Prescribing characteristics				
Geometric mean OME mgs per patient^a^	467 (351–665)	200 (153–478)	108 (82–146)	323 (225–478)
Geometric mean OME mgs per dispensing^a^	308 (251–388)	183 (140–240)	102 (79–135)	240 (175–338)
Total medicine classes prescribed, *n*	38 (32–42)	26 (19–34)	5 (4–8)	15 (10–21)
% dispensings for opioids	17% (14%–22%)	25% (17%–36%)	67% (53%–78%)	45% (23%–63%)
% opioid dispensing for codeine	21% (13%–29%)	43% (32%–55%)	32% (13%–59%)	5% (1%–11%)
% opioid dispensing for tramadol	12% (6%–20%)	13% (7%–22%)	1% (0%–7%)	0% (0%–3%)
% opioid dispensing for oxycodone	39% (30%–48%)	29% (19%–41%)	54% (30%–75%)	63% (50%–75%)
% opioid dispensing for morphine	2% (0%–4%)	0% (0%–1%)	0% (0%–0%)	6% (0%–13%)
% opioid dispensing for buprenorphine	8% (4%–16%)	1% (0%–4%)	0% (0%–0%)	1% (0%–6%)
% opioid dispensing for fentanyl	2% (0%–5%)	0% (0%–1%)	0% (0%–0%)	3% (0%–8%)
Characteristics of patients prescribed opioids				
Age in years, mean	62 (58–67)	51 (47–55)	50 (45–55)	66 (62–70)
No prior opioid use in past year, %	34% (27%–41%)	41% (34%–50%)	60% (48%–71%)	22% (15%–31%)
Only one opioid dispensing, %	49% (42%–57%)	72% (64%–79%)	92% (85%–98%)	65% (54%–75%)
Six or more opioid dispensings, %	20% (14%–28%)	5% (2%–9%)	0% (0%–0%)	0% (0%–6%)
Died, %	4% (2%–7%)	0% (0%–2%)	0% (0%–0%)	20% (9%–33%)
Cancer, %	8% (6%–11%)	4% (0%–9%)	4% (6%–11%)	68% (15%–85%)
Hospitalised, %	44% (39%–50%)	37% (31%–44%)	61% (50%–74%)	84% (78%–90%)
Days spent in hospital, median	0 (0–1)	0 (0–0)	1 (0–2)	10 (6–15)
Surgical procedure (received general anaesthesia), %	17% (13%–21%)	15% (11%–19%)	36% (21%–50%)	33% (25%–43%)
Inpatient chronic pain diagnosis^b^, %	3% (2%–7%)	2% (0%–4%)	0% (0%–3%)	10% (6%–17%)
Inpatient back pain diagnosis^b^, %	5% (3%–7%)	3% (2%–6%)	1% (0%–6%)	12% (7%–18%)
Inpatient joint pain diagnosis^b^, %	13% (10%–18%)	7% (5%–11%)	8% (3%–16%)	20% (13%–32%)
Prescribed antidepressants, %	25% (19%–33%)	12% (7%–18%)	0% (0%–0%)	3% (0%–8%)
Prescribed benzodiazepines, %	16% (11%–22%)	8% (4%–13%)	0% (0%–0%)	3% (0%–7%)
Prescribed NSAIDs, %	19% (13%–26%)	14% (8%–22%)	4% (0%–11%)	0% (0%–5%)
Prescribed pregabalin, %	12% (8%–17%)	5% (3%–9%)	0% (0%–0%)	4% (0%–9%)

Abbreviations: IQR, interquartile range; NSAID, nonsteroidal anti‐inflammatory drugs; OME, oral morphine equivalents.

^a^ Geometric mean is the mean calculated on the log scale and represents the central tendency of a lognormal (skewed) distribution.

^b^ Diagnoses recorded during a hospitalisation only.

**FIGURE 2:**
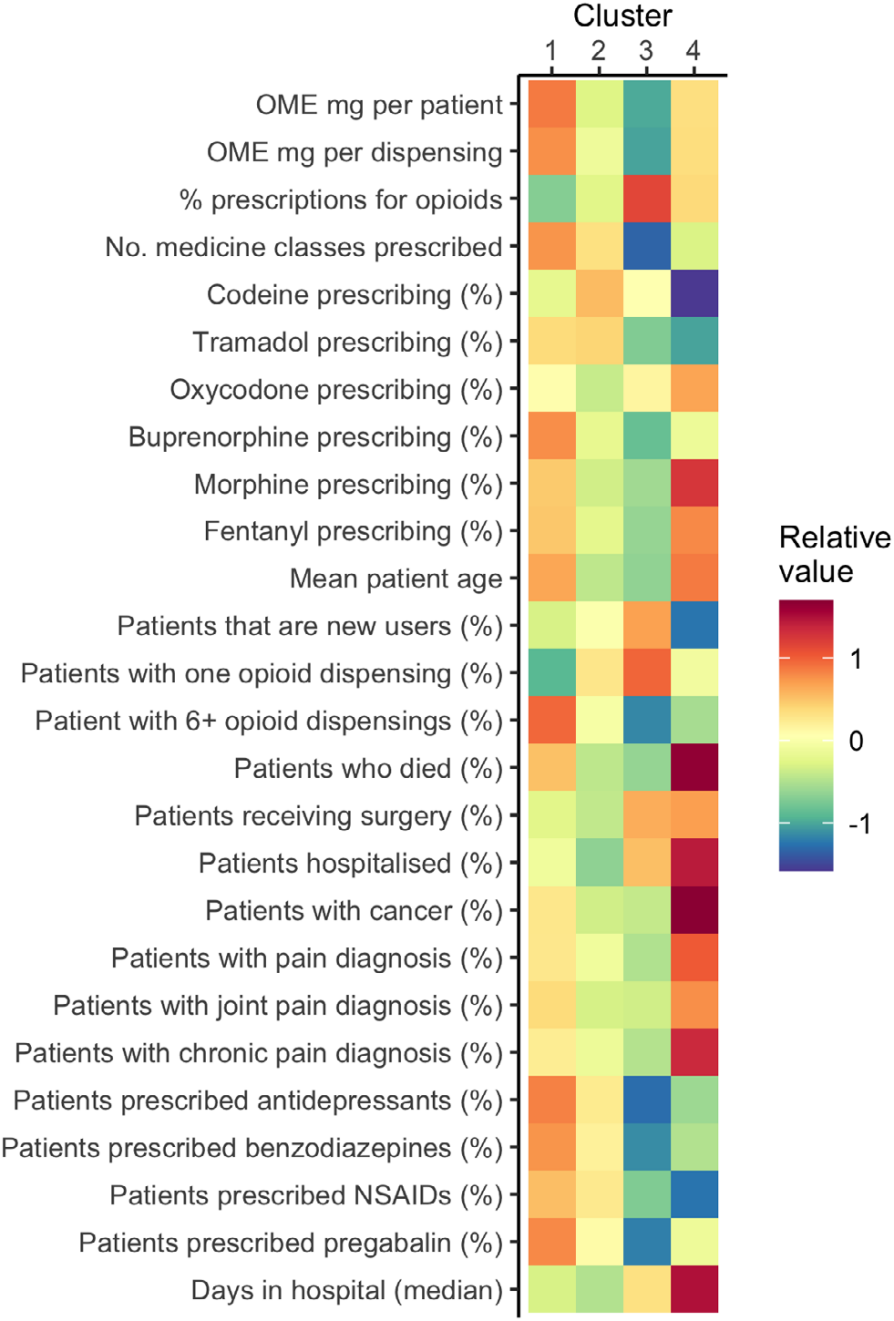
Heat map showing relative prevalence of each variable by cluster in 2018 (*n* = 14,874). Cluster 1 (*n* = 5536; 23.7%); Cluster 2 (*n* = 4387; 18.7%); Cluster 3 (*n* = 4013; 17.1%); Cluster 4 (*n* = 938; 4.0%). Red is higher prevalence, blue is lower prevalence. NSAID, nonsteroidal anti‐inflammatory drugs; OME, oral morphine equivalents.

Practitioners in the largest cluster (Cluster 1, *n* = 5536, 23.7%), prescribed opioids to older patients (median age 62 years, IQR 58–67) with high analgesic use and relatively fewer pain‐related comorbidities. This cluster also had the greatest percentage of patients prescribed other psychotropic and analgesic medicines (antidepressants [median = 25%], pregabalin [median = 12%], benzodiazepines [median = 16%] and nonsteroidal anti‐inflammatory drugs [median = 19%]), and a substantially greater percentage of patients with six or more opioid dispensings over the year (median = 20%) (Figure 2, Table 2). Practitioners in this cluster prescribed the greatest OME mgs per patient and more commonly prescribed buprenorphine compared with practitioners in other clusters.

Practitioners in the second largest cluster (Cluster 2, *n* = 4387, 18.7%) prescribed a wide range of medicines, and had a large proportion of patients (median = 72%) to whom they prescribed opioids once; they predominantly prescribed codeine and oxycodone. The mean age of patients prescribed opioids was 51 years.

Practitioners in Cluster 3 (*n* = 4013, 17.1%) prescribed opioids to younger patients for acute use. Practitioners in this cluster had patients with the lowest mean age (50 years) and had the greatest percentage of patients with an inpatient procedure requiring general anaesthesia (median = 36%), the highest percentage of new opioid use (median = 92%) and prescribed fewer non‐opioid medicines. They also prescribed the lowest OME mgs per patient and mostly prescribed codeine, tramadol and oxycodone.

Practitioners in the smallest cluster (Cluster 4, *n* = 938, 4.0%) prescribed opioids to patients with more pain‐related comorbidities and/or for palliative care. They had patients with the highest mean patient age (66 years), and the greatest proportion of patients with cancer (median = 68%) and pain‐related diagnoses recorded during hospitalisation, specifically chronic pain (median = 10%), back pain (median = 12%) and joint pain (median = 20%). This cluster also had the greatest percentage of patients hospitalised (median = 84%) or who died in the year (median = 20%).

### 3.3 Opioid prescribing by practitioner cluster

While practitioners in Cluster 1 comprised 23.7% of practitioners, they prescribed 76.4% of OME mgs dispensed to the study population, to 71.1% of NSW residents dispensed opioids in 2018. One‐third of practitioners in this cluster were in the top 10% of practitioners based on OME mgs prescribed. In contrast, practitioners in Cluster 3 comprised 17.1% of practitioners but prescribed only 1.6% of OME mgs (Table 3). Cluster 2 comprised 18.7% of practitioners who prescribed opioids to nearly half (47.2%) of the study population.

**TABLE 3:** Opioid prescribing by cluster and in practitioners with fewer than 10 patients who were not included the cluster analysis in 2018 (*n* = 23,408).

	Cluster 1	Cluster 2	Cluster 3	Cluster 4	<10 patients^b^
Opioid prescribers, *n* (%)	5536 (23.7%)	4387 (18.7%)	4013 (17.1%)	938 (4.0%)	8534 (36.5%)
OME grams, *n* (%)	1,728,896 (76.7%)	419,752 (18.6%)	35,024 (1.6%)	51,680 (2.3%)	19,559 (0.9%)
Opioid dispensings, *n* (%)	2,584,179 (69.2%)	812,169 (21.7%)	222,226 (6.0%)	71,804 (1.9%)	45,885 (1.2%)
Opioid patients^a^, *n* (%)	560,503 (71.1%)	371,692 (47.2%)	192,214 (24.4%)	38,407 (4.9%)	29,323 (3.7%)
In top 10% of prescribers by OME, *n* (%)	2027 (36.6%)	300 (6.8%)	<5	23 (2.5%)	<5

Abbreviation: OME, oral morphine equivalents.

^a^ Some patients may appear in multiple clusters and thus percentages add up to >100%; total number of patients = 788,205.

^b^ Not included in cluster analysis.

### 3.4 Sensitivity analyses

Table S6 displays the opioid prescribing distribution excluding practitioners without a dispensing in each quarter of the year. We observed similar results to the full population, with the proportion of OME mgs prescribed by the top centile (1%) of practitioners approximately 1% lower in all years (e.g., 13.9% in 2018). Table S7 shows the proportions in each practitioner cluster in 2016 and 2017. There was little variation, with similar proportions observed as in 2018 within 5%.

## 4 DISCUSSION

We quantified for the first time variation among medical practitioners in opioid prescribing in a representative Australian, whole‐of‐population cohort. We identified substantial differences in opioid prescribing across medical practitioners who commonly prescribe opioids, with the top centile prescribing many orders of magnitude more opioids than those in the bottom centiles, with a large proportion of practitioners prescribing very few opioids. These patterns were consistent over time.

There are few studies of opioid practitioner‐level prescribing in Australia, and therefore no data against which to benchmark our findings. One 2021 study quantified prescribing of opioid agonist treatment in NSW [19], while another study from Queensland found that most opioid prescribers primarily prescribed low doses [20]. However, our findings differ from other jurisdictions, such as the United States where the top 1% of prescribers prescribed 49% of OME mgs [4]. While the rates of opioid use and harms are lower than in North America, opioid consumption is still high and increasing [21]. The reasons for these differences are multifactorial, and may include differences in healthcare systems, restrictions on direct‐to‐consumer advertising, and availability of harm reduction programs such as opioid agonist treatment for opioid dependence [22, 23]. Like other countries Australia has implemented a range of regulatory interventions and other responses to minimise harms, with varying degrees of success, although unlike the United States most states currently lack prescription drug monitoring programs [24].

We identified four distinct clusters characterising practitioners based on their opioid prescribing patterns and characteristics of their patients who had their opioid prescriptions dispensed. People likely to be undergoing palliation often require strong pain relief, and this is one area where there is evidence that opioids are effective, especially among people with advanced cancer [25, 26]. However, opioid prescribing for these patients represented only a small proportion of the total OME mgs dispensed; the population at the end‐of‐life represent a minority of people prescribed opioids [27], although likely to require high doses over a relatively short period of time.

We have described overall patterns, but were not able to determine appropriate or inappropriate prescribing, especially in the light of the lack of prescriber specialty data. However, we did identify prescribing practices that may be concerning. We found a high volume of opioids prescribed by practitioners for older patients with both high amounts of both opioid and non‐opioid analgesic use; a large proportion of patients were dispensed six or more opioid prescriptions over 1 year, and one in six were prescribed benzodiazepines and one in eight pregabalin, both of which should be used with caution when combined with opioids due to the increased risk of harms [28, 29]. There is a lack of high‐quality evidence for use of opioids for treating chronic non‐cancer pain, especially long‐term, and careful consideration should be made when prescribing for this indication [30, 31]. Although practitioners in this cluster only comprised 24% of the entire practitioner population in this study, they prescribed at least one opioid to 71% of people in NSW dispensed PBS‐listed opioids in 2018. Opioid prescribing to older patients, especially long‐term and in combination with other sedating medicines, should be done with caution, due to increased risk of falls and other adverse effects [32] and several Australian studies have identified substantial inappropriate use of opioids in Australian aged care [33, 34].

Prescriber practices represent only one factor impacting opioid use, which is also highly variable at the patient‐level [35], and some patients receive prescriptions from multiple practitioners [36]. While some interventions—such as real time prescription monitoring—target patients, many interventions designed to improve opioid prescribing specifically target practitioners, such as increasing restrictions on prescribing or prescriber education [24]. Yet, the top prescribers in our study prescribed a very large amount of all opioids and represented a minority of prescribers, suggesting that more targeted interventions or different approaches may be required.

### 4.1 Strength and limitations

In this unique study population, we had data on all adults in NSW who were prescribed and dispensed a PBS‐listed opioid during the study period, meaning that we captured all opioids prescribed by NSW practitioners. A limitation of this study is that data on practitioner specialty was not available in our data, which would help further explain variations in prescribing; for instance, pain and palliative care specialists may have high rates of opioid prescribing which are appropriate due to the types of patients they see. Previous studies have found that general practitioners are large prescribers of opioids, and are responsible for half of all opioid initiations [37]. However, this is one of first Australian studies to describe practitioner‐level prescribing which can form a basis for further studies on the impact of prescriber specialty. We also did not have information on practice size; practitioners who prescribed opioids to a large number of patients may simply have a greater practice size overall.

Further, we have not captured all opioid prescribing, and are missing data on opioids that are prescribed privately, or in public hospitals, and opioids prescribed but not dispensed. However, in general practice, private prescriptions account for only 6% of all opioid prescriptions [38]. Opioid agonist treatment for opioid dependence was also not included, but in 2017 there were only 1010 active opioid agonist treatment prescribers with most having fewer than five clients [19]. Lastly, we have used the partitioning around medoids algorithm, but there are several different approaches to clustering, which may find slightly different results. However, all clustering methods should be viewed as approximations, and patterns using different methods tend to identify broadly similar patterns [16, 17].

## 5 CONCLUSION

We quantified the distribution of opioid prescribing by practitioners in an Australia population‐based sample, providing a benchmark for future research on opioid prescribing behaviour in this jurisdiction. While a large proportion of practitioners prescribed few opioids to a small number of patients, there was substantial variation with the top centile practitioners who prescribed opioid amounts many‐fold higher than the lowest deciles. While such variation in prescribing is not unexpected, there is a need to better understand opioid prescriber characteristics as targeted interventions, as opposed to broad‐brush restrictions on prescribing, may be more helpful in improving prescribing practices. Further investigation into how patient factors contribute to practitioner behaviour to drive opioid prescribing is also warranted.

## AUTHOR CONTRIBUTIONS

Each author certifies that their contribution to this work meets the standards of the International Committee of Medical Journal Editors.

## CONFLICT OF INTEREST STATEMENT

In 2020, the Centre for Big Data Research in Health, UNSW Sydney received funding from AbbVie Australia to conduct post‐market surveillance research. AbbVie did not have any knowledge of, or involvement in, the current study. In the past 3 years, Louisa Degenhardt has received funding from Indivior, Seqirus for studies of new opioid medicines in Australia. Sallie‐Anne Pearson and Andrew Wilson are members of the Drug Utilisation Sub Committee of the Pharmaceutical Benefits Advisory Committee. The views expressed in this paper do not necessarily represent those of the Committee.

## Supporting information


**Data S1.** Supporting information.
