# Supplementary material for: Opioid prescribing patterns among medical practitioners in New South Wales, Australia
Source: Drug Alcohol Rev. 2023 May 9;42(6):1472–81. doi: 10.1111/dar.13675 (PMC10946566; doi:10.1111/dar.13675)
Supplement: Supplementary file 1 — Data S1. Supporting information. [file DAR-42-1472-s001.docx]

**Supporting Information**

**Figure S1.** Flow chart of selection of New South Wales (NSW)-based practitioners who prescribed opioids to NSW residents, 2013-2018.


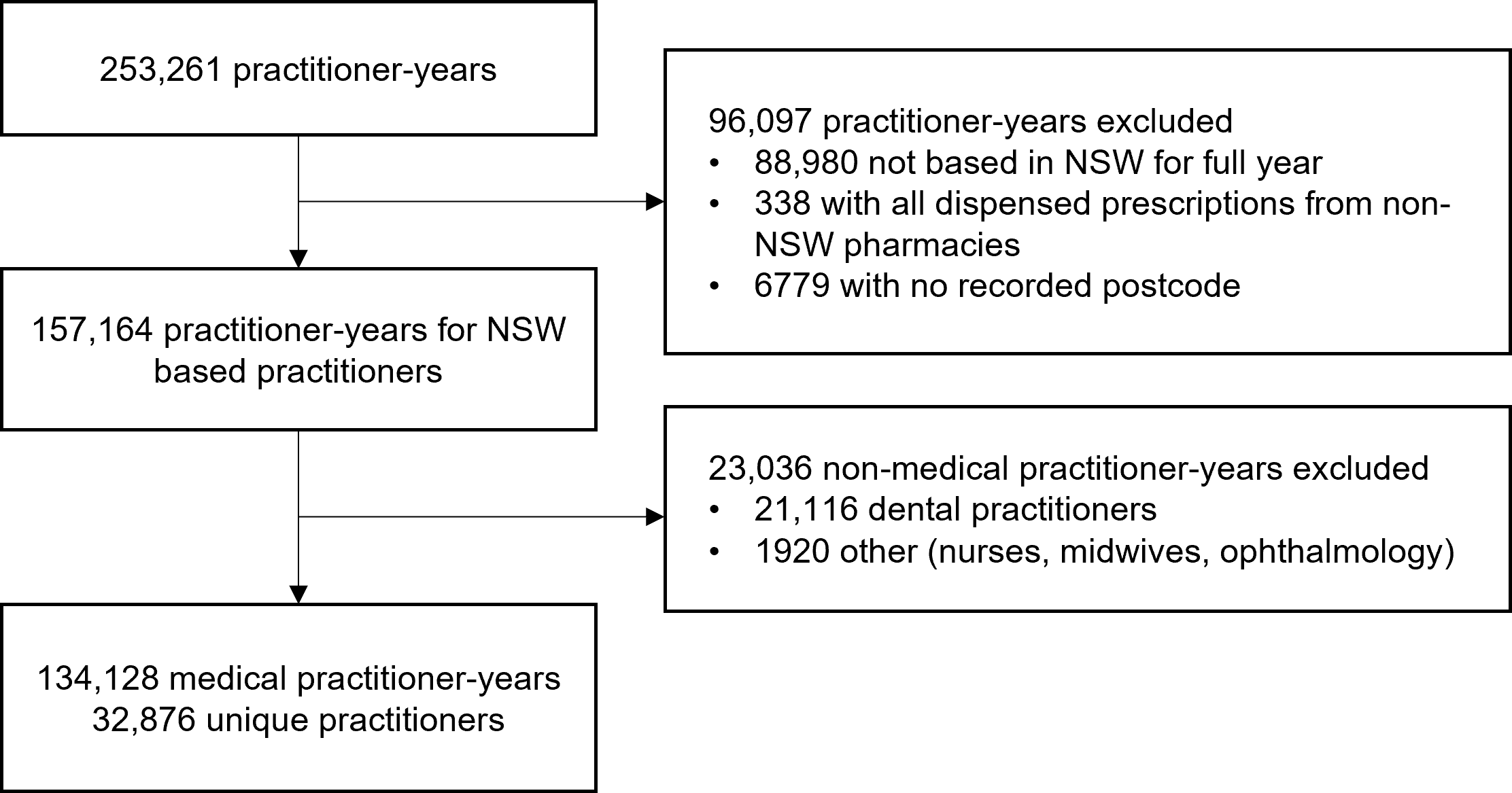


**Table S1.** Definition of variables included in clustering analysis of prescribers in 2018 and overall summary statistics. All variables measured among people dispensed opioids only in 2018.

| List of variables | Data source | Definition |
| --- | --- | --- |
| Geometric mean OME per patient | PBS | Geometric mean OME mgs prescribed to each patient in 2018 |
| Geometric mean OME per dispensing | PBS | Geometric mean OME mgs prescribed per dispensing |
| % opioid dispensings for codeine | PBS | $\frac{\text{No. codeine dispensings}}{\text{No. opioid dispensings}}\times\text{100}$ |
| % opioid dispensings for buprenorphine | PBS | $\frac{\text{No. buprenorphine dispensings}}{\text{No. opioid dispensings}}\times\text{100}$ |
| % opioid dispensings for tramadol | PBS | $\frac{\text{No. tramadol dispensings}}{\text{No. opioid dispensings}}\times\text{100}$ |
| % opioid dispensings for oxycodone | PBS | $\frac{\text{No. oxycodone dispensings}}{\text{No. opioid dispensings}}\times\text{100}$ |
| % opioid dispensings for morphine | PBS | $\frac{\text{No}\text{.}\text{ morphine dispensings}}{\text{No. opioid dispensings}}\times\text{100}$ |
| % opioid dispensings for fentanyl | PBS | $\frac{\text{No. fentanyl dispensings}}{\text{No. opioid dispensings}}\times\text{100}$ |
|  |  |  |
| % patient initiating opioids | PBS | $\frac{\text{No. opioid initiators (new opioid dispensing using a 365 day lookback period)}}{\text{No. patients }}\times\text{100}$ |
| % patients with one opioid dispensing only | PBS | $\frac{\text{No. patients with one opioid dispensing}}{\text{No. opioid dispensings}}\times\text{100}$ |
| % dispensing that are opioids | PBS | $\frac{\text{No. opioid dispensings}}{\text{No. dispensings}}\times\text{100}$ |
|  |  |  |
| Mean patient age | PBS | Mean age at first dispensing |
| % patients died | NDI | $\frac{\text{No. patients with death record in 2018}}{\text{No. patients }}\times\text{100}$ |
| % patients with cancer | APDC, PBS | $\frac{\text{No. patients with cancer}}{\text{No. patients }}\times\text{100}$  Cancer defined as inpatient diagnosis of ICD-10-AM code C or ≥1 dispensing of WHO ATC code L01, L02 |
| % patients with back pain diagnosis recorded during hospitalisation | APDC | $\frac{\text{No. patients with a back pain diagnosis}}{\text{No. patients }}\times\text{100}$  Back pain defined as an inpatient diagnosis of ICD-10-AM code M40-M54 (“Dorsopathies”) |
| % patients with joint pain diagnosis recorded during hospitalisation | APDC | $\frac{\text{No. patients with a joint pain diagnosis}}{\text{No. patients}}\times\text{100}$  Joint pain defined as inpatient diagnosis of ICD-10-AM code M00-M25 (“Arthropathies”), U86.2 (“Arthritis and osteoarthritis”) |
| % patients with chronic pain diagnosis recorded during hospitalisation | APDC | $\frac{\text{No. patients with a chronic pain diagnosis}}{\text{No. patients}}\times\text{100}$  Chronic pain defined as inpatient diagnosis of ICD-10-AM code R52.1 (“Chronic intractable pain”), R52.2 (“Other chronic pain”) |
| % patients hospitalised | APDC | $\frac{\text{No. patients with ≥1 hospital sep}\text{a}\text{ration}}{\text{No. patients}}\times\text{10}\text{0}$ |
| Median patient length of stay | APDC | Median total days patients spent in hospital in 2018 |
| % patients with surgery | APDC | $\frac{\text{No. patients with general anaesthesia procedure}}{\text{No. patients}}\times\text{100}$  General anaesthesia defined as inpatient ACHI procedure code 92514 |
| % patients prescribed antidepressants | PBS | $\frac{\text{No. patients also prescribed antidepressants (ATC code N06A)}}{\text{No. patients }}\times\text{100}$ |
| % patients prescribed benzodiazepines | PBS | $\frac{\text{No. patients also prescribed benzodiazepines (ATC code N05BA,N05CD)}}{\text{No. patients }}\times\text{100}$ |
| % patients prescribed pregabalin | PBS | $\frac{\text{No. patients also prescribed pregabalin}}{\text{No. patients }}\times\text{100}$ |
| % patients prescribed NSAID | PBS | $\frac{\text{No. patients also prescribed antidepressants (ATC code M01A)}}{\text{No. patients }}\times\text{100}$ |
| Total no. medicine classes prescribed | PBS | Number of unique 3-digit ATC classes prescribed |

ACHI, Australian Classification of Health Interventions; APDC, New South Wales Admitted Patients Data Collection; ATC, Anatomic Therapeutic Classification; ICD-10-AM, International Classification of Diseases 10^th^ edition Australian Modification; NDI, National Death Index; NSAID, non-steroidal anti-inflammatory drug; OME, oral morphine equivalent; PBS, Pharmaceutical Benefits Scheme; WHO, World Health Organization.

**Table S2.** Number of registered medical practitioners in New South Wales (<https://www.ahpra.gov.au/Publications/Annual-reports/Annual-report-archive.aspx>)

| **Year** | **No. practitioners** |
| --- | --- |
| 2013/14 | 31,269 |
| 2014/15 | 32,183 |
| 2015/16 | 33,236 |
| 2016/17 | 34,235 |
| 2017/18 | 35,303 |
| 2018/19 | 36,194 |

**Table S3.** Number of practitioners and total opioids prescribed by year

| **Year** | **No. practitioners** | **No. OME mg prescribed per practitioner, median (IQR)** | **No. patients prescribed opioids per practitioner, median (IQR)** |
| --- | --- | --- | --- |
| 2013 | 20179 | 7959 (840-99863) | 20 (4-73) |
| 2014 | 21530 | 7972 (850-97005) | 21 (4-74) |
| 2015 | 22502 | 8010 (930-95327) | 20 (4-72) |
| 2016 | 22758 | 8410 (930-93897) | 21 (4-70) |
| 2017 | 23751 | 7820 (878-87743) | 20 (4-68) |
| 2018 | 23410 | 8557 (900-86963) | 21 (4-70) |

IQR, interquartile range; OME, oral morphine equivalent.

**Table S4.** Total oral morphine equivalents dispensed by top 1%, 10% and 50% of practitioners

|  | **Top 1%** | **Top 10%** | **Top 50%** |
| --- | --- | --- | --- |
| 2013 | 15.2% | 63.6% | 99.2% |
| 2014 | 15.4% | 63.9% | 99.2% |
| 2015 | 15.6% | 64.4% | 99.2% |
| 2016 | 15.8% | 64.3% | 99.1% |
| 2017 | 16.1% | 64.9% | 99.2% |
| 2018 | 15.1% | 63.9% | 99.1% |

**Table S5.** Key distinguishing features of practitioner groups in practitioners with 10+ patients (n=14,874)

| **Cluster** | **Prescribing characteristics** | **Characteristics of patients prescribed opioids** |
| --- | --- | --- |
| Cluster 1(n=5536) | - Highest OME mgs per patient and per dispensing - Prescribe a wide range of medicines - Most commonly prescribe oxycodone, codeine | - Older age - High proportion of patients with >=6 opioid dispensings in a year - Highest rates of prescribing of antidepressants, benzodiazepines, NSAIDs and pregabalin |
| ” Cluster 2 (n=4387) | - Moderate OME mgs per patient and dispensing - Prescribe wide range of medicines - Most commonly prescribe codeine, oxycodone | - Younger age - High rates of patients with only 1 opioid dispensing - Moderate rate of prescribing of other analgesic medicines |
| Cluster 3 (n=4013) | - Lowest OME mgs per patient and per dispensing - Prescribe mostly opioids - Most commonly prescribe oxycodone, codeine | - Youngest age - Very high proportion of patients with only 1 opioid dispensing - Highest rate of inpatient procedures requiring anesthesia - Very low prescribing of other analgesic medicines |
| Cluster 4(n=938) | - High OME mgs per patient and per dispensing - Most commonly prescribe oxycodone - Highest rates of prescribing of morphine | - Oldest age - Very high rates of patients with cancer - Highest rates of diagnoses of chronic pain, back pain, joint pain - Highest rates of patients hospitalised |

OME, oral morphine equivalent; NSAID, nonsteroidal anti-inflammatory drugs.

**Figure S2.** Lorenz curve showing the distribution of OME mgs prescribed in, 2013-2018.The dashed red lines show the proportion of OME mgs prescribed by the top 50%, 90%, and 99% of practitioners. If opioid prescribing were equally distributed among practitioners, the solid line would be straight with a slope of 1. OME, oral morphine equivalent.


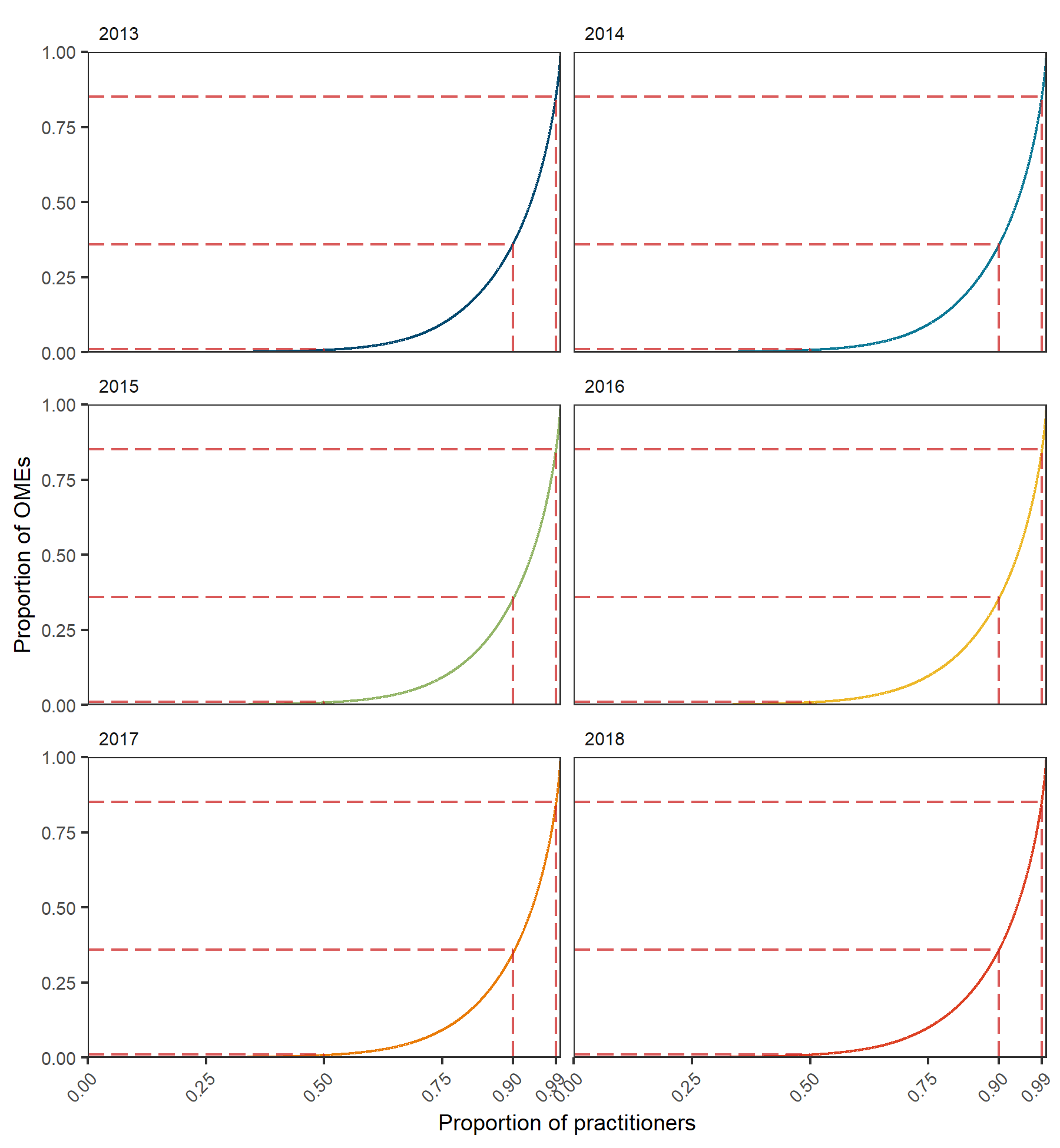


**Sensitivity analyses**

**Table S6.** Total OMEs dispensed by top 1%, 10% and 50% of prescribers excluding prescribers without a dispensing in each quarter

|  | **Top 1%** | **Top 10%** | **Top 50%** |
| --- | --- | --- | --- |
| 2013 | 13.9% | 59.7% | 98.7% |
| 2014 | 14.0% | 59.9% | 98.7% |
| 2015 | 14.2% | 60.4% | 98.7% |
| 2016 | 14.5% | 60.5% | 98.6% |
| 2017 | 14.7% | 60.9% | 98.7% |
| 2018 | 13.9% | 60.5% | 98.6% |

**Table S7.** Number of practitioners in each cluster, 2016-2018

|  | **2016** | **2017** | **2018** |
| --- | --- | --- | --- |
|  | **N (%)** | **N (%)** | **N (%)** |
| **Cluster 1** | 4921 (21.6%) | 4428 (18.6%) | 5536 (23.7%) |
| **Cluster 2** | 4496 (19.8%) | 4902 (20.6%) | 4013 (17.1%) |
| **Cluster 3** | 4154 (18.3%) | 4461 (18.8%) | 4387 (18.7%) |
| **Cluster 4** | 902 (4.0%) | 1123 (4.7%) | 938 (4.0%) |
| **<10 patients** (not included in the cluster analysis) | 8285 (36.4%) | 8837 (37.2%) | 8534 (36.4%) |
